# Should evoked potential monitoring be used in degenerative cervical spine surgery? A systematic review

**DOI:** 10.1186/s10195-019-0524-4

**Published:** 2019-04-02

**Authors:** Alberto Di Martino, Rocco Papalia, Antonio Caldaria, Guglielmo Torre, Luca Denaro, Vincenzo Denaro

**Affiliations:** 1 Department of Orthopaedic and Trauma Surgery, University Campus Bio-Medico of Rome, Via Alvaro Del Portillo, 200, 00128 Rome, Italy; 2 Sidney Kimmel Medical College of Thomas Jefferson University (SKMC), Philadelphia, USA; 3 Department of Neurosurgery, University of Padova, Padua, Italy

**Keywords:** SSEP, MEP, IONM, Evoked potentials, Cervical spine surgery

## Abstract

**Background:**

Intraoperative somatosensory evoked potential (SSEP) and transcranial motor evoked potential (tcMEP) monitoring are frequently used in spinal as well as spinal cord surgery for so-called intraoperative neuromonitoring (IONM), while the combination of these techniques is known as concomitant multimodal intraoperative monitoring (MIOM). The aim of this review is to collect available evidence concerning use of IONM and MIOM in cervical decompression surgery in the degenerative setting and attempt to identify the best practice to be advocated.

**Materials and methods:**

A review of the PubMed and MEDLINE databases and Cochrane Central Registry of Controlled Trials was performed. Studies were included if they involved patients who underwent cervical spine decompression surgery for degenerative stenosis with use of IONM or MIOM and where sensitivity/specificity was reported.

**Results:**

In the identified studies, the sensitivity of SSEP was estimated to be between 22 and 100% with constant specificity of 100%. In the included studies, the sensitivity of MEP was estimated to be between 78 and 100% with specificity ranging from 83.2 to 100%.

**Conclusions:**

On the basis of available evidence, MIOM could be a helpful tool in decompression cervical spine surgery in patients affected by degenerative spinal stenosis, since it is associated with high specificity and sensitivity for detection of intraoperative neural damage. However, evidence is still lacking regarding patient selection to identify individuals in whom monitoring is indicated.

**Level of evidence:**

IV (systematic review of studies with LOE II to IV).

## Introduction

Cervical spine decompression is a surgical procedure aimed at relieving pressure on neural elements as well as reducing pain caused by neural impingement. Decompression surgery is performed as primary treatment for cervical spinal stenosis, herniated disc, cervical injuries including fractures, epidural/intradural extramedullary tumors, as well as spinal cord tumors and other expanding diseases. It has been reported that cervical radiculopathy has an annual incidence of 107.3 per 100,000 for men and 63.5 per 100,000 for women, with a peak at 50–54 years of age. A more recent study conducted among US military personnel found an incidence of 1.79 per 1000 person-year [27]. An epidemiological study based on the Nationwide Inpatient Sample demonstrated that 1,323,979 cervical spine surgical procedures were performed in the USA between 2002 and 2009 [27]. Anterior cervical discectomy and fusion (ACDF) was the most frequent surgical procedure performed in that time period, accounting for 80.3% of all surgical procedures for cervical decompression. Posterior cervical decompression (PCD) and posterior cervical fusion (PCF) accounted for 11.2% and 8.5% of procedures, respectively [21].

When performed in the degenerative setting, decompression of neural elements can be achieved using direct or indirect procedures [19, 20]. Direct decompression is achieved by resection of the impinging bone, ligaments, and disc material that are directly compressing the neural elements [10]. Indirect decompression procedures can be divided into segmental and global spinal alignment procedures [3, 4].

Segmental procedures are mainly performed by distraction between two vertebrae, which leads to opening of the neural foramen and an increase in epidural space. Global spinal alignment procedures are associated with decompression laminectomy and allow the spinal cord to migrate dorsally away from areas of anterior compression [6].

Spinal decompression is a functional surgery; therefore, mainly in the degenerative setting, any neurological worsening after surgery is an unexpected complication. Intraoperative neurophysiological monitoring (IONM) is widely applied in spinal surgery to prevent such postoperative neurological worsening. From a historical perspective, IONM was used for the first time in the 1970s by Dr. Brown to reduce the risk of damage to the spinal cord, albeit during surgery for scoliosis [5]. The targets of neurophysiological monitoring in spinal surgery are somatosensory evoked potentials (SSEPs) and motor evoked potentials (MEPs). SSEP monitoring is used to identify changes in sensory pathways, which run through the posterior columns of the spinal cord. SSEP monitoring yields a plot that reflects the sequential activation of the neural structures along somatosensory pathways. Transcranial motor evoked potential (tcMEP) monitoring is used to identify potential damage to motor pathways, which pass through lateral and anterior–lateral columns of the spinal cord. TcMEPs are electrical responses recorded either from muscles or from axons of the descending motor tract in response to electrical or magnetic stimulation of nervous system structures that control movement. IONM represents a tool to assess spinal cord functional integrity, allowing early detection and reversal of neurological sensory or motor deficits. IONM can therefore be considered to be a specific monitoring tool for neurophysiological pathways and has became an ideal intraoperative real-time neuromonitoring procedure that warrants routine use in spine surgery. Multimodal intraoperative monitoring (MIOM) is a combination of these techniques and enables full assessment of the whole spinal cord function.

SSEP monitoring is extensively used in spinal deformity corrective surgery, e.g., in patients affected by scoliosis or kyphosis. Use of IONM in this setting has been studied extensively, since it can be considered the only alternative to the Stagnara wake-up test [33]. Even though its use in deformity surgery has become the standard for this procedure, use of IONM during cervical spine decompression surgery in the degenerative setting remains controversial [29]. Therefore, the main aim of this study is to collect available evidence concerning IONM for either motor or somatosensory potentials in cervical decompression surgery in patients affected by degenerative spinal stenosis. Furthermore, an attempt is made to evaluate the role of use of these techniques in combination.

## Materials and methods

The present work was carried out in accordance with Preferential Reporting Items for Systematic Reviews and Meta-analyses (PRISMA) guidelines.

### Eligibility criteria

Only peer-reviewed publications were considered for inclusion. Studies were included if they involved patients who underwent cervical spine decompression surgery with IONM, using SSEP and/or tcMEP, and that reported their sensitivity and specificity. All studies had to assess a population composed of adults. The types of study considered for inclusion were randomized controlled trials (RCTs), case series (CS), retrospective case series (RCS), and prospective cohort studies (PCS), while case reports, literature reviews, and meta-analyses were excluded. According to the reviewers’ language capabilities, considered studies were those written in English, Italian, Spanish, and French. Studies on animals, in vitro or biomechanical studies, and cadaver experiments were also excluded.

### Information sources and search

Electronic research to identify eligible studies was performed using online databases including PubMed–MEDLINE and the Cochrane Central Registry of Controlled Trials by two reviewers (A.D.M. and A.C). The literature search was carried out in the period from March to July 2017. The search strings utilized were (“evoked potentials”[MeSH Terms] OR (“evoked”[All Fields] AND “potentials”[All Fields]) OR “evoked potentials”[All Fields] OR (“evoked”[All Fields] AND “potential”[All Fields]) OR “evoked potential”[All Fields]) AND (“spine”[MeSH Terms] OR “spine”[All Fields]) AND (“surgery”[Subheading] OR “surgery”[All Fields] OR “surgical procedures, operative”[MeSH Terms] OR (“surgical”[All Fields] AND “procedures”[All Fields] AND “operative”[All Fields]) OR “operative surgical procedures”[All Fields] OR “surgery”[All Fields] OR “general surgery”[MeSH Terms] OR (“general”[All Fields] AND “surgery”[All Fields]) OR “general surgery”[All Fields]).

### Study selection

**Fig. 1:**
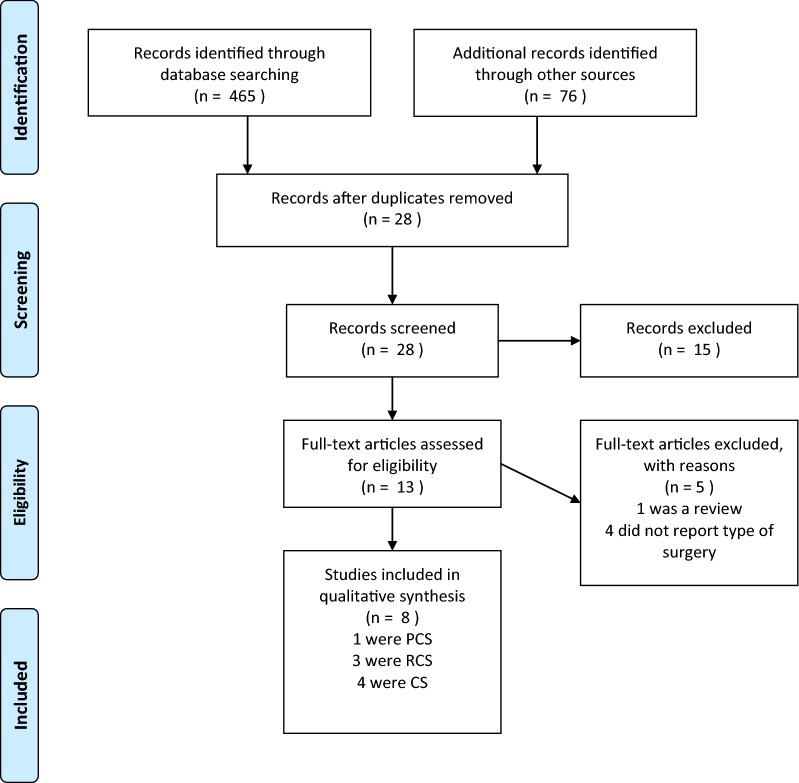
PRISMA 2009 flow diagram (from Ref. [18])

Once the studies eligible for inclusion had been retrieved, the full text of articles was obtained and evaluated. A manual search through the bibliography of each of the relevant articles was also performed to identify potentially missed eligible papers. Duplicates were removed. The study selection process, carried out in accordance with the PRISMA flowchart, is shown in Fig. 1.

### Study quality and risk of bias of the studies

The quality of the studies was evaluated according to American Academy of Orthopaedic Surgeons (AAOS) clinical practice guidelines and systematic review methodology [31]. The following questions are used to evaluate the quality of diagnostic study designs: Was the patient spectrum representative of the patients who will receive the test in practice? Were the selection criteria clearly described? Was the execution of the index and reference tests described in sufficient detail to permit its replication? Is the reference standard likely to correctly classify the target condition? Are the index test results interpreted by an examiner without knowledge of the reference tests results? A study is considered as high quality if it has < 1 flaw, as moderate quality if it has ≥ 1 and < 2 flaws, as low quality if it has ≥ 2 and < 3 flaws, and as very low quality if it has ≥ 3 flaws. The risk of bias was determined according to the type of study available. The assessment of the risk of bias was performed using the Risk of Bias in Nonrandomized Studies of Intervention (ROBINS-I) tool [30].

### Data collection process

All the included studies were analyzed, and the following data were extracted and are summarized in Table 1: study type and level of evidence, type of procedure and type of monitoring used, MEP changes, SSEP changes, sensitivity and specificity of testing, onset of new neurological deficits, and numbers of false-positive and false-negative cases. A false positive was defined as presence of SSEP and/or tcMEP warnings during positioning or the surgical procedure that was not followed by clinically ascertained neurological deficits in the postoperative period. A false negative was defined as the absence of warnings during IONM or recovery of SSEPs or MEPs after repositioning, but with the occurrence of new neurological deficits after surgery. According to literature concerning the procedure, the sensitivity was calculated as true positives/(true positives + false negatives), while the specificity was calculated as true negatives/(true negatives + false positives) [1].

**Table 1:** Details of included studies

Study	Year	Type of study	Level of evidence	No. of recruited patients	M	F	Type of monitoring	Mean age at intervention (years)
				Tot = 1683	Tot = 576	Tot = 383		
				Av = 240.43	Av = 115.2	Av = 76.6	–	Av = 57.8
				SD = 152.03	SD = 78.09	SD = 70.87	–	SD = 5.2
Plata Bello et al.	2015	CS	IV	75	53	22	TcMEP SSEP	60
Appel et al.	2017	CS	IV	381	–	–	TcMEP	–
Hilibrand et al.	2004	PCS	II	427	242	185	TcMEP SSEP	–
Sakaki et al.	2012	CS	IV	357	–	–	TcMEP	–
Oya et al.	2017	RCS	IV	135	91	40	MEP SSEP	62
Garcia et al.	2010	RCS	IV	80	56	24	SSEP	61
Xu et al.	2011	RCS	IV	57	–	–	TcMEP SSEP	48
Eggspuehler et al.	2007	CS	IV	246	134	112	SSEP	58

*M* male, *F* female, *Av* average, *SD* standard deviation, *RCS* retrospective case study, *PCS* prospective case study, *tcMEP* transcranial motor evoked potential, *SSEP* somatosensory evoked potential, *MIOM* multimodal intraoperative monitoring

## Results

### Included studies

According to the search performed, a total of eight studies met the inclusion criteria and were included for review. Of these studies, one was a PCS [13], four were CS [2, 8, 24, 26], and three were RCS [11, 22, 35]. The studies included in the search reported data on a total of 1683 patients. According to AAOS clinical practice guidelines and systematic review methodology, six of the included studies were rated as high quality, while two were considered to be of moderate quality. The risk of bias judgements according to ROBINS-I are presented in Table 2. Five studies were evaluated to have moderate overall risk of bias, while three studies had an overall serious risk of bias.

**Table 2:** Risk of Bias Judgements in Nonrandomized Studies of Interventions (ROBINS-I) evaluations

Study	Confounding	Selection of participants	Classification of interventions	Deviations from intended interventions	Missing data	Measurement of outcomes	Selection of reported results	Overall
Plata Bello et al.	Serious	Moderate	Moderate	Serious	Serious	Moderate	Moderate	Serious
Appel et al.	Low	Low	Low	Low	Moderate	Low	Moderate	Moderate
Hilibrand et al.	Moderate	Moderate	Low	Low	Low	Low	Low	Moderate
Sakaki et al.	Serious	Serious	Moderate	Serious	Moderate	Moderate	Moderate	Serious
Oya et al.	Serious	Serious	Moderate	Serious	Moderate	Moderate	Moderate	Serious
Garcia et al.	Low	Low	Moderate	Moderate	Moderate	Low	Low	Moderate
Xu et al.	Low	Moderate	Low	Low	Low	Moderate	Moderate	Moderate
Eggspuehler et al.	Moderate	Moderate	Low	Low	Moderate	Moderate	Low	Moderate

### Type of surgery

All patients evaluated in the included studies underwent surgical decompression of the cervical spine. In the included studies, anterior, posterior, and combined approaches were performed in equal fractions, accounting for 33% each. However, two studies did not report any data concerning surgical approach.

### Types of monitoring

Types of monitoring included in our search were SSEP, MEP, and the combination thereof (MIOM). Two studies included patients monitored only by SSEP [8, 11], two studies included patients monitored only by MEP [2, 26], four studies included patients monitored by SSEP and MEP separately [13, 22, 24, 35], and one study included patients monitored by concomitant MIOM [35].

### Sensitivity and specificity

In the included studies, the sensitivity of SSEP was estimated to be between 22% [2] and 100% [11] with constant specificity of 100% [13, 14, 22, 24, 26, 35]. The sensitivity of MEP reported in the studies included in our search was estimated to be between 78% [2] and 100% [13, 14, 22, 24, 26, 35] with specificity ranging from 83.2% [26] to 100% [13, 17, 24]. Three studies [8, 11, 35] included in our search reported cases of false positives and false negatives during SSEP and MEP monitoring. In general, MEP was more sensitive and specific than SSEP, while combined use of MEP and SSEP showed better results compared with their separate use (Table 3). Furthermore, SSEP monitoring showed more false-positive and false-negative results than MEP monitoring (Table 4).

**Table 3:** Sensitivity and specificity of intraoperative monitoring

Study name	Year	No. of patients	IOM change	MEP change	SSEP change	Sensitivity and specificity (SSEP)	Sensitivity and specificity (MEP)	No. of new neurological deficits
Plata Bello et al.	2015	75	5 (6.6%)	5	2	(40%; 100%)	(100%; 100%)	–
Appel et al.	2017	381	9 (2.3%)	7	2	(22%; 100%)	(78%; 100%)	2
Hilibrand et al.	2004	427	15 (3.5%)	12	3	(25%; 100%)	(100%; 100%)	2
Sakaki et al.	2012	357	196 (55%)	196	–	–	(100%; 83.2%)	0
Oya et al.	2017	135	12 (8.9%)	12	0	–	(100%; 98.4%)	–

*IOM* intraoperative monitoring, *SSEP* somatosensory evoked potential, *MEP* motor evoked potential

**Table 4:** False negatives and false positives of intraoperative monitoring

Study name	Year	No. of patients	False negatives	False positives	Sensitivity and specificity
Garcia et al.	2010	80	SSEP = 0	SSEP = 1	(100%; 99%)
Xu et al.	2011	57	SSEP = 2, MEP = 0	SSEP = 2, MEP = 1	(33%; 95,6%) (100%; 98%)
Eggspuehler et al.	2007	246	SSEP = 2	SSEP = 2	(83%; 99%)

*IOM* intraoperative monitoring, *SSEP* somatosensory evoked potential, *MEP* motor evoked potential

### Postoperative neurological deficits

The most frequent postoperative deficit reported in the included studies was nerve root injury [8], followed by unilateral upper limb motor and sensory deficit [11]. Presented data suggest that intraoperative changes in evoked potential monitoring correlate with the onset of clinically ascertained deficits in the postoperative period. The neurological complication rate in the studies included in our search was 2.17%, including both motor and sensory deficits (Table 5).

**Table 5:** Clinically assessed postoperative deficits

Name	Year	No. of patients	New postoperative deficits	Types of deficit
Appel et al.	2016	381	2	–
Hilibrand et al.	2004	427	2	1 paraplegia, 1 upper extremity paraplegia
Sakaki et al.	2012	357	0	–
Garcia et al.	2010	80	4	3 unilateral upper extremity motor and sensory deficits, 1 complete spinal cord injury
Eggspuehler et al.	2007	246	12	11 nerve root injury, 1 quadriplegia

## Discussion

The present study focused on a comparison of the outcomes, sensitivity, and specificity of SSEP and MEP, used in combination or separately in patients affected by degenerative cervical spinal stenosis. Limitations of this study are the heterogeneity of the studies included in the search, and the fact that only a limited number of studies reported the surgical approach. For this reason, it was not possible to analyze the sensitivity and specificity of evoked potential monitoring in relation to the surgical approach. Furthermore, the preoperative MEP and SSEP of the patients and the site of monitoring were not reported, and therefore not analyzed.

Concerning the level of evidence, no randomized clinical trials were retrieved by the literature search on this topic. The review included one PCS, four CS, and three RCS. The risk of bias of the included studies, assessed using the ROBINS-I tool, was evaluated as moderate to serious, thus the conclusions of those studies must be considered with substantial caution when analyzing the findings of the studies included in this systematic review (Table 2).

The types of surgical procedure performed in the included studies did not reflect the global literature context [21], where the anterior approach is most commonly performed. Unfortunately, the heterogeneity of the approaches used in the included studies prevented accurate comparison of results. In literature, in contrast to our results, other authors have reported lower sensitivity values for SSEP, ranging from 0 to 52%, and higher specificity, ranging from 95 to 100% [12, 23, 25, 28]. Other studies, in contrast to our results, have reported higher sensitivity values for MEP, ranging from 75 to 100%, and the same specificity range from 84 to 100% [23, 28]. Although muscle registered MEPs are considered the gold standard for detection of new postoperative motor deficits, multimodality monitoring has become the standard practice for a variety of spinal procedures, and several reported studies have shown that SSEP combined with MEP has sensitivity and specificity approaching 100% [7, 25]. A study reported a statistically significant difference in the sensitivity and specificity of MEP when total signal loss was taken into account or other alarm criteria were considered [32]. The site of monitoring is a key factor, influencing signal loss; muscles with multiple-level innervation may show partial signal loss when one level is altered or damaged. Therefore, partial signal lost may also be an alarm criterion, depending on the muscle tested [2]. This feature is not reported and well discussed in most of the papers, therefore results may be misinterpreted. Moreover, use of partial signal loss is applied in some cases to allow the surgeon to detect partial alterations during surgery, avoiding further damage [2, 22]. The threshold of potential considered is also relevant for monitoring. It was suggested by Appel et al. [2] that low thresholds may lead to false-positive cases, since low-level signal losses may represent subclinical damage to nerve tracts, not elicited by physical examination. Another possible explanation for the occurrence of false positives is the position of the patient during surgery. In some reported cases, it was observed that a significant reduction or abolishment of abnormalities registered during IONM occurred after repositioning the patient at the end of surgery [2]; moreover, clinical experience reveals that some cases of alterations of potentials may already occur during the positioning of the patient, suggesting that IONM should begin before patient positioning, and end when the patient is awakened.

Another electrophysiological feature of IONM that is relevant to damage assessment is the pattern of signal loss. It has been suggested that sudden signal loss is associated with severe and irreversible injury, while more gradual degeneration is more likely to represent mild, recoverable damage. Wang et al. recently investigated this aspect [34], reporting that a pattern of gradual loss was observed more frequently during cervical spine procedures, with a sudden loss for thoracic spinal procedures. However, most of the studies did not report specific data about damage patterns, thus complete evaluation of this feature in the included studies is not possible. Since clinical reversibility of damage has been associated with progressive signal loss, this could be a determining factor in defining false positives. Concerning the clinical assessment of the neurological state, a recent report by Khan et al. [15] showed that the incidence of new postoperative neurological deficits after cervical spine decompression surgery was 2.4%. In the evaluation presented herein, nerve root injuries were the most frequent postoperative neurological deficits. A study reported that the incidence of C5 root deficits for the anterior and posterior approach in decompression at C4–C5 level was 12% [9]. C5 palsy was commonly caused by iatrogenic injury, reperfusion injury of the spinal cord, or impingement by an osteophyte with a tethering effect. However, IONM can only detect intraoperative neurophysiological changes, while neurological deficits may occur immediately afterwards. Therefore, the occurrence of delayed C5 palsy makes it difficult to evaluate the efficacy of IONM [16].

The base hypothesis advocated in this study is that the combination of SSEP and MEP might be more sensitive and specific than IONM. However, according to the results of this review, this hypothesis cannot be confirmed, due to lack of data concerning combined sensitivity and specificity. On the basis of available evidence, we support use of MIOM in decompression cervical spine surgery in patients affected by degenerative spinal stenosis, since it is associated with high specificity and sensitivity for detection of intraoperative neural damage. Furthermore, we support the idea that IONM should begin before positioning of the patient in the operating room. However, given the lack of appropriate evidence, we recommend that better and more focused studies be carried out to clarify whether the combination of SSEP and MEP is more sensitive and specific than either method alone. Furthermore, evidence concerning appropriate selection of patients in whom monitoring is indicated is still lacking, and this should be a focus of future studies on this topic.

## Abbreviations

LOE: level of evidence
IONM: intraoperative neurologic monitoring
MEP: motor evoked potential
SSEP: somatosensory evoked potential
MIOM: multimodal intraoperative monitoring
CS: case series
PCS: prospective cohort study
RCS: retrospective case series
